# 2018 WSES/SIS-E consensus conference: recommendations for the management of skin and soft-tissue infections

**DOI:** 10.1186/s13017-018-0219-9

**Published:** 2018-12-14

**Authors:** Massimo Sartelli, Xavier Guirao, Timothy C. Hardcastle, Yoram Kluger, Marja. A. Boermeester, Kemal Raşa, Luca Ansaloni, Federico Coccolini, Philippe Montravers, Fikri M. Abu-Zidan, Michele Bartoletti, Matteo Bassetti, Offir Ben-Ishay, Walter L. Biffl, Osvaldo Chiara, Massimo Chiarugi, Raul Coimbra, Francesco Giuseppe De Rosa, Belinda De Simone, Salomone Di Saverio, Maddalena Giannella, George Gkiokas, Vladimir Khokha, Francesco M. Labricciosa, Ari Leppäniemi, Andrey Litvin, Ernest E. Moore, Ionut Negoi, Leonardo Pagani, Maddalena Peghin, Edoardo Picetti, Tadeja Pintar, Guntars Pupelis, Ines Rubio-Perez, Boris Sakakushev, Helmut Segovia-Lohse, Gabriele Sganga, Vishal Shelat, Michael Sugrue, Antonio Tarasconi, Cristian Tranà, Jan Ulrych, Pierluigi Viale, Fausto Catena

**Affiliations:** 1 Department of Surgery, Macerata Hospital, Macerata, Italy; 2 Unit of Endocrine, Head, and Neck Surgery and Unit of Surgical Infections Support, Department of General Surgery, Parc Taulí Hospital Universitari, Sabadell, Spain; 3 Trauma Service, Inkosi Albert Luthuli Central Hospital and Department of Surgery, Nelson R Mandela School of Clinical Medicine, Durban, South Africa; 4 Department of General Surgery, Division of Surgery, Rambam Health Care Campus, Haifa, Israel; 5 Department of Surgery, Academic Medical Centre, Amsterdam, Netherlands; 6 Department of Surgery, Anadolu Medical Center, Kocaali, Turkey; 7 General Surgery Department, Bufalini Hospital, Cesena, Italy; 8 Anesthesiology and Critical Care Medicine, Paris Diderot Sorbonne Cite University, Bichat-Claude Bernard University Hospital, HUPNSV, Paris, France; 9 Department of Surgery, College of Medicine and Health Sciences, UAE University, Al-Ain, United Arab Emirates; 10 Infectious Diseases Unit, Department of Medical and Surgical Sciences, Alma Mater Studiorum University of Bologna, Bologna, Italy; 11 Infectious Diseases Division, Department of Medicine University of Udine and Azienda Sanitaria Universitaria Intergrata di Udine, Udine, Italy; 12 Trauma and Acute Care Surgery, Scripps Memorial Hospital La Jolla, La Jolla, CA USA; 13 General Surgery-Trauma Team, State University of Milano, Niguarda Hospital Milano, Milan, Italy; 14 Emergency Surgery Unit, State University of Pisa, Cisanello Hospital, Pisa, Italy; 15 Riverside University Health System Medical Center and Loma Linda University School of Medicine, Moreno Valley, CA USA; 16 Department of Medical Sciences, Infectious Diseases, University of Turin, Turin, Italy; 17 Unit of General, Emergency and Trauma Surgery, Regional Hospital of Perpignan, Perpignan, France; 18 Department of Surgery, Addenbrooke’s Hospital, Cambridge University Hospitals NHS Foundation Trust, Cambridge, UK; 19 Second Department of Surgery, Aretaieion University Hospital, National and Kapodistrian University of Athens, Athens, Greece; 20 Department of Emergency Surgery, City Hospital, Mozyr, Belarus; 21 Global Alliance for Infections in Surgery, Porto, Portugal; 22 Abdominal Center, University Hospital Meilahti, Helsinki, Finland; 23 Department of Surgical Disciplines, Immanuel Kant Baltic Federal University, Regional Clinical Hospital, Kaliningrad, Russian Federation; 24 Department of Surgery, University of Colorado, Denver Health Medical Center, Denver, CO USA; 25 Department of Surgery, Emergency Hospital of Bucharest, Bucharest, Romania; 26 Infectious Diseases Unit, Bolzano Central Hospital, Bolzano, Italy; 27 Department of Anesthesia and Intensive Care, Azienda Ospedaliero-Universitaria Parma, Parma, Italy; 28 Department of Surgery, UMC Ljubljana, Ljubljana, Slovenia; 29 Department of General and Emergency Surgery, Riga East University Hospital ‘Gailezers’, Riga, Latvia; 30 General Surgery Department, Colorectal Surgery Unit, La Paz University Hospital, Madrid, Spain; 31 General Surgery Department, Medical University, University Hospital St George, Plovdiv, Bulgaria; 32 Second Department of Surgery, Hospital de Clínicas, Universidad Nacional de Asuncion, San Lorenzo, Paraguay; 33 Emergency Surgery (or Division of Emergency Surgery), Fondazione Policlinico Universitario A. Gemelli IRCCS – Università Cattolica del Sacro Cuore, Rome, Italy; 34 General Surgery, Tan Tock Seng Hospital, Singapore, Singapore; 35 Department of Surgery, Letterkenny University Hospital and Donegal Clinical Research Academy, Letterkenny, Ireland; 36 Department of Emergency Surgery, Parma Maggiore Hospital, Parma, Italy; 37 First Department of Surgery, Department of Abdominal, Thoracic Surgery and Traumatology, First Faculty of Medicine, Charles University and General University Hospital, Prague, Czech Republic

**Keywords:** Soft-tissue infections, Necrotizing infection, Surgical site infection

## Abstract

Skin and soft-tissue infections (SSTIs) encompass a variety of pathological conditions that involve the skin and underlying subcutaneous tissue, fascia, or muscle, ranging from simple superficial infections to severe necrotizing infections. SSTIs are a frequent clinical problem in surgical departments. In order to clarify key issues in the management of SSTIs, a task force of experts met in Bertinoro, Italy, on June 28, 2018, for a specialist multidisciplinary consensus conference under the auspices of the World Society of Emergency Surgery (WSES) and the Surgical Infection Society Europe (SIS-E). The multifaceted nature of these infections has led to a collaboration among general and emergency surgeons, intensivists, and infectious disease specialists, who have shared these clinical practice recommendations.

## Introduction

Skin and soft-tissue infections (SSTIs) encompass a variety of pathological conditions that involve the skin and underlying subcutaneous tissue, fascia, or muscle, ranging from simple superficial infections to severe necrotizing infections. SSTIs may affect any part of the body and are a frequent clinical problem in surgical departments [1, 2].

Successful management of patients with severe SSTIs involves prompt recognition, appropriate antibiotic therapy, timely surgical debridement or drainage, and resuscitation when required.

Several critical issues have been debated in the management of these patients. In order to clarify these major issues in the management of SSTIs, a panel of experts met in Bertinoro, Italy, on June 28, 2018, for a specialist multidisciplinary consensus conference under the auspices of the World Society of Emergency Surgery (WSES) and the Surgical Infection Society Europe (SIS-E).

During the consensus conference, 17 panelists presented the statements developed for each of the main questions regarding the diagnosis and management of SSTIs. An agreement on all the statements was reached.

The expert panel met via email to prepare and revise the consensus paper resulting from the meeting. The manuscript was successively reviewed by all members and ultimately revised as the present manuscript. This document represents the executive summary of the consensus conference which outlines clinical recommendations based on the Grading of Recommendations Assessment, Development, and Evaluation (GRADE) hierarchy criteria summarized in Table 1 [3].

**Table 1:** Grading of Recommendations Assessment, Development and Evaluation (GRADE) hierarchy of evidence from Guyatt et al. [3]

Grade of recommendation	Clarity of risk/benefit	Quality of supporting evidence	Implications
1A			
Strong recommendation, high-quality evidence	Benefits clearly outweigh risk and burdens, or vice versa	RCTs without important limitations or overwhelming evidence from observational studies	Strong recommendation, applies to most patients in most circumstances without reservation
1B			
Strong recommendation, moderate-quality evidence	Benefits clearly outweigh risk and burdens, or vice versa	RCTs with important limitations (inconsistent results, methodological flaws, indirect analyses, or imprecise conclusions) or exceptionally strong evidence from observational studies	Strong recommendation, applies to most patients in most circumstances without reservation
1C			
Strong recommendation, low-quality or very low-quality evidence	Benefits clearly outweigh risk and burdens, or vice versa	Observational studies or case series	Strong recommendation but subject to change when higher quality evidence becomes available
2A			
Weak recommendation, high-quality evidence	Benefits closely balanced with risks and burden	RCTs without important limitations or overwhelming evidence from observational studies	Weak recommendation, best action may differ depending on the patient, treatment circumstances, or social values
2B			
Weak recommendation, moderate-quality evidence	Benefits closely balanced with risks and burden	RCTs with important limitations (inconsistent results, methodological flaws, indirect, or imprecise) or exceptionally strong evidence from observational studies	Weak recommendation, best action may differ depending on the patient, treatment circumstances, or social values
2C			
Weak recommendation, low-quality or very low-quality evidence	Uncertainty in the estimates of benefits, risks, and burden; benefits, risk, and burden may be closely balanced	Observational studies or case series	Very weak recommendation; alternative treatments may be equally reasonable and merit consideration

## How should SSTIs be classified?

**The term “skin and soft-tissue infections” describes a wide heterogeneity of clinical conditions. We recommend that the necrotizing or non-necrotizing character of the infection, the anatomical extension, the characteristics of the infection (purulent or not purulent), and the clinical condition of the patient should be always assessed independently to classify patients with soft-tissue infections (recommendation 1C).**

SSTIs encompass a variety of pathological conditions involving the skin and underlying subcutaneous tissue, fascia, or muscle and ranging from simple superficial infections to severe necrotizing infections.

Various classification systems have been used to describe SSTIs including variables such as anatomic location, causative pathogen(s), rate of progression, depth of infection, and severity of clinical presentation.

In 1998, the US Food and Drug Administration (FDA) classified SSTIs into two broad categories for the purpose of clinical trials evaluating new antimicrobials for their treatment: uncomplicated and complicated. Uncomplicated SSTIs included superficial infections such as cellulitis, simple abscesses, impetigo, and furuncles and required antibiotics or surgical incision for drainage of abscess alone. In contrast, complicated SSTIs included deep soft-tissue infections such as necrotizing infections, infected ulcers, infected burns, and major abscesses which required significant surgical intervention with drainage and debridement [4].

The terms “complicated” and “uncomplicated” persist and can be useful in describing SSTIs as reported by Napolitano [5].

Uncomplicated SSTIs carry low risk for life- or limb-threatening infection unless they are improperly treated. Patients who have uncomplicated SSTIs can be treated with either empiric antibiotic therapy according to the most probable pathogen and local resistance patterns in impetigo, erysipelas, or mild cellulitis with drainage and debridement or simple surgical drainage in skin abscess.

Complicated SSTIs are associated with high risk for life-threatening infection. In patients who have complicated SSTIs, it is of paramount importance to initiate appropriate and adequate broad-spectrum initial empiric antibiotic therapy and to consider the need for surgical intervention for drainage and/or debridement.

In 2003, Eron et al. [6] classified SSTIs according to the severity of local and systemic signs and the presence or absence of comorbid conditions in patients presenting in the outpatient setting to guide the clinical management, treatment, and admission decisions. In this classification system, SSTIs were divided in four classes:

- Class 1: patients with SSTI, but no signs or symptoms of systemic toxicity or co-morbidities.
- Class 2: patients are either systemically unwell with stable co-morbidities or are systemically well, but have a comorbidity (e.g., diabetes, obesity) that may complicate or delay resolution.
- Class 3: patients appear toxic and unwell (fever, tachycardia, tachypnoea, and/or hypotension).
- Class 4: patients have sepsis syndrome and life-threatening infection; for example necrotizing fasciitis.

SSTIs may be also classified according to the anatomical tissue layers involved [7]. Superficial infections such as erysipelas, impetigo, folliculitis, furuncles, and carbuncles are located at the epidermal and dermal layer, while cellulitis is located in the dermis and subcutaneous tissue. Deep infections extend below the dermis and may involve the subcutaneous tissue, fascial planes, or muscular compartments presenting as complex abscesses, fasciitis, or myonecrosis.

Complicated SSTIs may also be classified as non-necrotizing or necrotizing infections. Necrotizing infections most commonly involve the muscular fascial layers but may also involve the dermal, subcutaneous, and muscle layers and warrant prompt, aggressive surgical debridement.

In 2014, the Infectious Diseases Society of America (IDSA) updated practice guidelines for the diagnosis and management of skin and soft-tissue infections [8]. The guidelines divided infections by purulent and non-purulent, severity (mild, moderate, and severe), and tissue necrosis (necrotizing versus non-necrotizing).

Recently, the US FDA has introduced the new definition of acute bacterial skin and skin-structure infection (ABSSSI) to more closely define complicated soft-tissue infection for the purposes of registration trials. ABSSSIs include cellulitis/erysipelas, wound infections, and major cutaneous abscesses. Thus, an ABSSSI is defined as a bacterial infection of the skin with a lesion size area of ≥ 75 cm^2^ (lesion size measured by the area of redness, edema, or induration) [9].

In 2015, the WSES published its guidelines for management of SSTIs [10] proposing a new definition dividing SSTIs in three main groups: surgical site infections (SSIs), non-necrotizing SSTIs, and necrotizing SSTIs. SSIs are classified into two subgroups: incisional and organ and organ/space. The incisional SSIs are further divided into superficial (skin and subcutaneous tissue) and deep (deep soft-tissue muscle and fascia). Organ and organ/space infections are not truly soft-tissue infections. Non-necrotizing SSTIs including erysipelas, impetigo, folliculitis, simple abscess, and complex abscess may be treated by antibiotics or drainage alone.

Necrotizing SSTIs (cellulitis, fasciitis, myositis, Fournier’s gangrene) require surgical intervention including drainage and debridement of necrotic tissue in addition to antibiotic therapy.

Several authors and organizations have proposed classification schemes for SSTIs based on such variables as anatomic location, rate of progression, depth of extension, and clinical presentation or severity. Each has key limitations both in assisting clinical management and in providing guidance for developing new therapeutic agents.

The consensus concluded that the necrotizing or non-necrotizing character of the infection, the anatomical extension, the characteristics of the infection (purulent or non-purulent), and the clinical conditions of the patient should be always assessed independently to classify patients with soft-tissue infections.

## What is new in the prevention of SSIs? What are the principles of SSI prevention?

**Recent global guidelines for the prevention of SSIs can support healthcare workers to develop or strengthen infection prevention and control programs, with a focus on surgical safety, as well as antimicrobial resistance action plans. We recommend that all healthcare workers adopt these evidence-based recommendations in their clinical practice (recommendation 1C).**

SSIs are the most common healthcare-associated infections among surgical patients. It is obviously important to improve patient safety by reducing the occurrence of SSIs. Preventing SSIs is a global priority. Bacteria are becoming increasingly resistant to antibiotics, making SSI prevention even more important nowadays.

SSIs are a major clinical problem in terms of morbidity, mortality, length of hospital stay, and overall direct and not-direct costs worldwide. Despite progress in prevention knowledge, SSIs remain one of the most common adverse events in hospitals. SSI prevention is complex and requires the integration of a range of measures before, during, and after surgery.

Both the World Health Organization (WHO) [11, 12] and the Centers for Disease Control and Prevention (CDC) [13] have recently published guidelines for the prevention of SSIs. The 2016 WHO Global guidelines for the prevention of surgical site infection [11, 12] are evidence-based including systematic reviews presenting additional information in support of actions to improve practice.

The guidelines include 13 recommendations for the pre-operative period, and 16 for preventing infections during and after surgery. They range from simple precautions such as ensuring that patients bathe or shower before surgery, appropriate way for surgical teams to clean their hands, guidance on when to use prophylactic antibiotics, which disinfectants to use before incision, and which sutures to use.

The proposed recommendations are as follows:

- “Strong” – Expert panel was confident that benefits outweighed risks, considered to be adaptable for implementation in most (if not all) situations, and patients should receive intervention as course of action.
- “Conditional” – Expert panel considered that benefits of intervention probably outweighed the risks; a more structured decision-making process should be undertaken, based on stakeholder consultation and involvement of patients and healthcare professionals.

Importantly, the guidelines recommend that antibiotic prophylaxis should be used to prevent infections before and during surgery only. Antibiotics should not be used after surgery, as is often done. Antibiotic prophylaxis should be administered for operative procedures that have a high rate of postoperative surgical site infection, or when foreign materials are implanted. Antibiotic prophylaxis should be administered within 120 min prior to the incision. However, administration of the first dose of antibiotics is dependent on its pharmacological characteristics. Underlying patient factors may also affect drug disposition (e.g., malnourishment, obesity, cachexia, and renal disease with protein loss may result in suboptimal antibiotic exposure through increased antibiotic clearance in the presence of normal or augmented renal function). Additional antibiotic doses should be administered intraoperatively for procedures > 2–4 h (typically where duration exceeds two half-lives of the antibiotic). There is no evidence to support the use of postoperative antibiotic prophylaxis. The key evidence-based recommendations outlined in these guidelines should be adopted by all healthcare staff that care for surgical patients throughout all stages of that patient’s surgical care.

## What is the best treatment of incisional SSIs? When are antibiotics needed?

**Incisional SSIs require prompt and wide opening of the surgical incision. We recommend antibiotic therapy for incisional SSIs with any Systemic Inflammatory Response Syndrome criteria or signs of organ failure such as hypotension, oliguria, decreased mental alertness, or in immunocompromised patients (recommendation 1C).**

SSIs are generally classified according to CDC criteria [14]. SSIs are classified as superficial incisional infection, deep incisional infection, and organ space infection. Superficial incisional infections are the most common type of SSIs. Deep incisional and organ/space are the types of SSIs that cause the most morbidity. Organ space infections are not genuine soft-tissue infections.

Incisional SSIs are the results of several factors [15]. All surgical wounds are contaminated by bacteria, but only a minority actually develops clinical infection. Colonization occurs when the bacteria begin to replicate and adhere to the wound site. If the host’s immune response is not sufficient to eliminate or overcome the effects of the bacteria, infection occurs [16]. In most patients, infection does not develop because host defenses are efficient to eliminate colonizers at the surgical site; however, in some patients, host defenses fail to protect them from SSIs. It is well known that surgical trauma increases inflammatory response and counter-regulatory mechanisms. Such regulatory mechanism can decrease postoperative immune response, promoting SSIs.

The pathogens isolated from infections differ, primarily depending on the type of surgical procedure. In clean-contaminated or contaminated surgical procedures, the aerobic and anaerobic pathogens of the normal endogenous microflora of the surgically resected organ are the most frequently isolated pathogens. In clean surgical procedures, in which the gastrointestinal, gynecologic, and respiratory tracts have not been entered, *Staphylococcus aureus* from the exogenous environment or the patient’s skin flora is the usual cause of infection. Nevertheless, in some specific body areas such as the groin skin could also be colonized by enteric flora. Moreover, it is possible that procedures such as hip prosthesis or vascular bypass, performed on this anatomical region, might eventually be infected by Gram-negative bacteria.

Sganga et al. [17] have recently reported that the risk factors associated with SSIs caused by *Methicillin-resistant Staphylococcus aureus* (MRSA), identified using the Delphi method were patients from long-stay care facilities, a hospitalization within the preceding 30 days, Charlson score > 5 points, chronic obstructive pulmonary disease and thoracic surgery, antibiotic therapy with beta-lactams (especially cephalosporins), and carbapenems and/or quinolones in the preceding 30 days, age 75 years or older, current duration of hospitalization > 16 days, and surgery with prosthesis implantation.

An important determinant of SSI is the integrity of host defenses. Important host factors include the following [18]: age, malnutrition status, diabetes, smoking, obesity, colonization with microorganisms, length of hospital stay or previous hospitalization, shock and hypoxemia, and hypothermia.

It is a common practice to cover surgical wounds with a dressing. The dressing acts as a physical barrier to protect the wound from contamination from the external environment until the wound becomes impermeable to microorganisms.

Postoperative care bundles recommend that surgical dressings be kept undisturbed for a minimum of 48 h after surgery unless leakage occurs. However, there are currently no specific recommendations or guidelines regarding the type of surgical dressing [19].

The diagnosis of incisional surgical site infection is clinical. Symptoms may include localized erythema, induration, warmth, and pain at the incision site. Purulent wound drainage and separation of the wound may occur. Most patients have systemic signs of infection such as fever and leukocytosis. Information on the microbiological species present in the wound is useful for determining antibiotic choice and predicting response to treatment.

An incisional SSI should be sampled if there is a clinical suspicion of infection. Lack of standardized criteria for diagnostic microbiology of SSIs present a challenge to monitor the global epidemiology of surgical site infection. Emergence of antibiotic resistance has made the management of SSIs difficult. Moreover, rapidly emerging nosocomial pathogens and the problem of multidrug resistance necessitates periodic review of isolation patterns and their sensitivity.

Adequate treatment of incisional SSIs should always include:

- Surgical incision and drainage of abscess.
- Debridement of necrotic tissue, if present.
- Appropriate wound care.
- Resuscitation to improve perfusion when sepsis is present.
- Adequate empiric antibiotic therapy when indicated.
- De-escalation when antibiogram is available.

Incisional SSIs should always be drained, irrigated, and if needed, opened and debrided. If fascial disruption is suspected, drainage should always be performed. Percutaneous drainage, wound irrigation, and negative pressure-assisted wound management are new and effective options that reduce the need for open management of wound infections. In cases where open management is needed, once the infection has cleared, the wound can be closed.

Superficial incisional SSIs that have been opened can usually be managed without antibiotics.

In patients with incisional SSIs with the presence of any systemic inflammatory response criteria or signs of organ failure such as hypotension, oliguria, decreased mental alertness, or in immunocompromised patients, empiric broad-spectrum antibiotic treatment should be started with coverage for Gram-positive cocci and/or the expected flora at the site of operation. Definitive antibiotic treatment is guided by the clinical response of the patient and, when available, results of gram stain, wound culture, and antibiogram.

## What is the appropriate treatment of superficial infections (impetigo, erysipelas and cellulitis, and superficial abscesses)?

**We recommend that impetigo, erysipelas, and cellulitis should be managed by antibiotics against Gram-positive bacteria (recommendation 1C).**

**Empiric therapy for community-acquired MRSA (CA-MRSA) should be recommended for patients at risk for CA-MRSA or who do not respond to first line therapy (recommendation 1C).**

**Incision and drainage is the primary treatment for simple abscesses or boils. We recommend not to use antibiotics for simple abscesses or boils (recommendation 1C).**

Superficial infections encompass either superficial spreading infection and inflammation within the epidermis and dermis that may be treated with antibiotics alone or a well-circumscribed abscess that may be treated by drainage alone.

Physical examination usually reveals erythema, tenderness, and induration. The majority of superficial SSTIs are caused by Gram-positive bacteria, particularly streptococci and *S. aureus*. The three common presentations of superficial infections consist of impetigo, erysipelas, and cellulitis. They are managed by antibiotic therapy against Gram-positive bacteria.

Impetigo is a highly contagious bacterial infection of the superficial layers of the epidermis. Impetigo predominantly affects children, and it is one of the most common SSTI in children worldwide [20]. It is characterized by discrete purulent lesions nearly always caused by β-hemolytic *Streptococcus* spp. and/or *S. aureus*. Moreover, of particular concern is the rising role of CA-MRSA as impetigo’s etiological agent [21–23].

Erysipelas is a fiery red, tender, painful plaque with well-demarcated edges and is commonly caused by streptococcal species, usually *Staphylococcus pyogenes. S. aureus* rarely causes erysipelas [24]. Streptococci are the primary cause of erysipelas. Most facial infections are attributed to group A *Streptococcus* (GAS), with an increasing percentage of lower extremity infections being caused by non-GAS. The role of *S. aureus*, and specifically MRSA, remains controversial [25].

Cellulitis is an acute bacterial infection of the dermis and the subcutaneous tissue that most commonly affects the lower extremities, although it can affect other areas. It causes local signs of inflammation, such as warmth, erythema, pain, lymphangitis, and frequently systemic upset with fever and raised white blood cell count [26]. As already reported in a previous paragraph, cellulitis has been recently classified as an ABSSSI together with erysipelas, SSIs, and major abscesses.

In a large European multicenter study, Garau et al. [27] analyzed a population of patients diagnosed with complicated SSTI hospitalized between December 2010 and January 2011 reporting that cellulitis was the most frequent diagnosis accounting for 59.1% of the total. Streptococci cause diffuse, rapidly spreading infection; staphylococcal cellulitis is typically more localized.

Treatment should begin promptly with agents effective against the typical Gram-positive pathogens, especially streptococci. If the cellulitis is very early and mild and no significant co-morbidities are present, oral beta-lactams might be sufficient in areas where CA-MRSA is not prevalent. Other available options are macrolides and lincosamides; however, resistance to erythromycin and clindamycin are increasing. Fluoroquinolones have been approved for the treatment of most uncomplicated cellulitis but are not adequate for treatment of MRSA infections. For more severe infections, parenteral route is the first choice.

If MRSA is suspected (both hospital acquired MRSA [HA-MRSA] and CA-MRSA), glycopeptides and newer antimicrobials are the best options [25, 28, 29]. For a simple superficial abscess or boil, incision and drainage is the primary treatment, and antibiotics are not needed. To be considered a simple abscess, induration and erythema should be limited only to a defined area of the abscess and should not extend beyond its borders. Additionally, simple abscesses do not extend into deeper tissues or have multiloculated extension. Cutaneous abscesses are typically caused by bacteria that represent the normal regional skin flora of the involved area [30].

## What is the appropriate treatment of complex abscesses (perianal and perirectal, and abscesses in intravenous drug injection sites)?

**Complex skin and subcutaneous abscesses are typically well circumscribed and respond to incision and drainage. We recommend antibiotic therapy if systemic signs of infection are present, in immunocompromised patients, if source control is incomplete or in cases of abscess with significant cellulitis (recommendation 1C).**

**We recommend empiric broad-spectrum antibiotic therapy with coverage of Gram-positive, Gram-negative, and anaerobic bacteria (recommendation 1C).**

Common sites of origin of complex abscesses may be perineal or perianal, perirectal, and abscesses at intravenous drug injection sites. Complicated skin and subcutaneous abscesses are typically well circumscribed and respond to incision and drainage with adjuvant antibiotic therapy.

Perianal and perirectal abscesses originate most often from an obstructed anal crypt gland, with the resultant pus collecting in the subcutaneous tissue, intersphincteric plane, or beyond (ischiorectal space or supralevator space) where various types of anorectal abscesses form. Once diagnosed, anorectal abscesses should be promptly drained surgically. An undrained anorectal abscess can continue to expand into adjacent spaces as well as progress to generalized systemic infection. Anorectal abscess occurs more often in males than females. Most patients present between the ages of 20 to 60 with the mean age of 40 in both sexes [31].

The diagnosis of anorectal abscess is usually based on the patient’s history and physical examination. The most common symptom of anorectal abscess is pain. As such, it has to be differentiated from other causes of anal pain including anal fissure, thrombosed hemorrhoids, levator spasm, sexually transmitted diseases, proctitis, and cancer. Low (intersphincteric, perianal, and ischiorectal) abscesses are usually associated with swelling, cellulites, and exquisite tenderness, but few systemic symptoms. High (submucosal, supralevator) abscesses may have few local symptoms, but significant systemic symptoms. Deeper abscesses, such as those that form in the supralevator or high ischiorectal space, may also present with pain referred to the perineum, low back, or buttocks.

The goal of surgical therapy of an abscess is to drain the abscess expeditiously, identify a fistula tract, and either proceed with primary fistulotomy to prevent recurrence or place a draining seton for future consideration.

A large abscess should be drained with multiple counter incisions rather than a long incision, which will create a step-off deformity and delay wound healing.

Complicated abscesses may involve a variety of pathogens and are frequently polymicrobial in origin.

Although most cases can be managed by incision and drainage, abscesses in injecting drug users require special considerations as compared to soft-tissue infections, which are not caused by intravenous drug abuse [32–35]. There are two main sources of organisms: the injecting drug users themselves (their oropharynx, skin, or feces), and the environment. Contamination may occur when the user prepares or injects the drug, uses shared needles, or re-uses injection paraphernalia. Manufacturing and handling of injectable drugs may be far from hygienic rules [36]. Persistent signs of systemic infection require evaluation for the presence of endocarditis. Foreign bodies, such as broken needles, should be ruled out by radiography, and duplex sonography should be performed to identify the presence of vascular complications [37]. Viral (HIV, HCV, HBV) acute or chronic infections should be always ruled out. Broad-spectrum antibiotics effective against aerobic and anaerobic organisms should be administered in patients with these infections. Broad-spectrum agents with coverage of Gram-positive, Gram-negative, and anaerobic pathogens may be required depending on the clinical setting. Given the high frequency of MRSA in some areas, this pathogen should be empirically covered if it is suspected, but no randomized studies are available for the treatment of SSTI specifically caused by CA-MRSA [1].

## What is the appropriate treatment of infections developing in damaged skin (burn wounds, animal and human bites, and pressure ulcers)?

**Irrigation of the wound and debridement of necrotic tissue are the most important factors in the prevention of infection and can substantially decrease the incidence of invasive wound infection. Antibiotic prophylaxis is not generally recommended (recommendation 1C).**

**For patients with systemic signs of infection, compromised immune status, severe comorbidities, associated severe cellulitis, severe and deep wounds, a broad-spectrum antibiotic effective against aerobic, and anaerobic organisms is always required (recommendation 1C).**

Infections developing in damaged skin are a heterogeneous group that includes bite wounds (animal and human bites), burn wounds, and pressure ulcers. If managed incorrectly, these infections can develop into more complicated soft-tissue infections.

Soft-tissue infection is the most common complication of animal and human bites. The risk of infection depends on the type of bite, the site of injury, the time elapsed from the bite until presentation, host factors, and the management of the wound [38–40]. In general, 10–20% of bite wounds become infected, including 30–50% of cat bites, 5–25% of dog bites, and 20–25% of human bites, respectively [41].

The predominant pathogens in these wounds are part of the normal oral flora of the biting animal, along with human skin organisms and occasional secondary invaders (e.g., *S. aureus* and GAS). Along with *Staphylococcus* ssp*.* (including MRSA) and *Streptococcus* ssp*.* (including *S. pyogenes*), the commonly isolated pathogens include *Pasteurella* spp*.* (*Pasteurella multocida*, *Pasteurella canis*, *Pasteurella dagmatis*), *Capnocytophagia canimorsus*, anaerobes (*Fusobacterium* spp*.*, *Prevotella* spp., *Bacteroides* spp., *Porphyromonas* spp.), and others. Streptococci can be isolated from 50% of human bite wounds, *S. aureus* from 40%, and *Eikenella corrodens* (a Gram-negative facultative anaerobic bacillus) from 30%. Human bites can transmit HBV, HCV, and HIV and post-exposure prophylaxis should be considered in every case [25].

There is clinical consensus that patients may be divided into low- and high-risk groups depending on the cause, nature, and location of the injury and on patient characteristics, but no evidence-based guidelines are currently available.

Deep irrigation of the wound serves to remove foreign bodies and pathogens. Irrigation under pressure is not recommended, as it may lead to the uncontrolled spread of bacteria into deeper tissue layers. Surgical treatment is based on the removal of necrotic tissue and mechanical reduction of the burden of pathogens. Universal prophylaxis with antibiotics is not recommended. The comprehensive meta-analyses of Medeiros et al. in the Cochrane Database [42] demonstrated no evidential basis for a reduction of the infection rate by prophylactic antibiotics, except for bite wounds on the hands. Despite the poor state of the evidence, most experts recommend early antibiotic treatment for 3 to 5 days for fresh, deep wounds and wounds in certain critical bodily areas (hands, feet, areas near joints, face, genitals), for persons at elevated risk of infection, and for persons with implants, such as artificial heart valves [43–45]. Antibiotics should not be given if the patient presents 24 h or more after the bite and there are no clinical signs of infection [46].

Significant burn injuries can predispose to infectious complications. Burn wound infections are one of the most important and potentially serious complications that occur in the acute period following injury. Accurate management of the wound with early excision of the eschar can substantially decrease the incidence of invasive burn wound infection. Damage to this barrier following a burn disrupts the innate immune system and increases susceptibility to bacterial infection. Although burn wound surfaces are sterile immediately following thermal injury, these wounds may be colonized with microorganisms. If the patient’s host defenses and therapeutic measures (such as excision of necrotic tissue and wound medications) are inadequate, microorganisms can colonize viable tissue, and a burn wound infection may occur.

Burn wound infections usually are polymicrobial. They can be immediately colonized by Gram-positive bacteria from the patient’s endogenous skin flora or the external environment. However, they can also be rapidly colonized by Gram-negative bacteria, usually within a week of the burn injury. Bacterial cultures can aid in the selection of an appropriate antibiotic, especially in cases of bacterial drug resistance, but altered pharmacokinetic parameters in burn patients must be considered and dosing should be adjusted accordingly to maximize antibiotic efficacy [47].

Pressure ulcers are localized areas of tissue necrosis that tend to develop when soft-tissue is compressed between a bony prominence and an external surface for a prolonged period of time. The damage may be relatively minor, or it may lead to massive destruction of deeper tissues. The majority of pressure ulcers develop in areas adjacent to the ischium, sacrum, and greater trochanter. Pressure ulcers represent a frequent problem especially in frail elderly patients with chronic co-morbidities [25]. When infection occurs, it is typically polymicrobial and includes aerobes (*S. aureus*, *Enterococcus* spp., *Proteus mirabilis*, *Escherichia coli*, *Pseudomonas* spp.) and anaerobes (*Peptococcus* spp., *Bacteroides fragilis*, *Clostridium perfringens*) [25].

Combination of surgical and antibiotic interventions may be required to manage infected decubitus ulcers. Surgical debridement is necessary to remove necrotic tissue. Antibiotic therapy should be used for patients with severe pressure ulcer infections, including those with spreading cellulitis or patients with systemic signs of infection. Because such infections usually are polymicrobial, therapeutic regimens should be directed against both Gram-positive and Gram-negative facultative organisms as well as anaerobic organisms. In many cases of pressure ulcers, correct wound care management can largely prevent the occurrence of these infections.

## When to administer antibiotics for MRSA in cSSTI?

**We recommend to administer antibiotics directed against MRSA as an adjunct to incision and drainage based on local epidemiology (area with more than 20% of MRSA in invasive hospital isolates or high circulation of MRSA in the community), specific risk factors for MRSA, and clinical conditions (recommendation 1C).**

The majority of SSTIs involving healthy skin are caused by aerobic Gram-positive cocci, specifically *S. aureus* and streptococci. Strains of *S. aureus* and GAS can produce a variety of toxins that may both potentiate their virulence and affect the soft tissues and allow invasion of the dermis. SSTIs management has recently become more complicated because of the increasing prevalence of multidrug-resistant pathogens.

Considerable variation in the resistance rates of *S. aureus* to methicillin (or oxacillin) in patients with SSTIs has been noted between continents, with the highest rates in North America (35.9%), followed by Latin America (29.4%) and Europe (22.8%) [48]. Although MRSA has been usually acquired during exposure in hospitals and other healthcare facilities, there has been a recent increase in MRSA infections presenting in the community (CA-MRSA) [49]. CA-MRSA strains are genetically and phenotypically distinct from HA-MRSA. CA-MRSA infections are becoming increasingly common. They can have a rapid and devastating course and may produce the pathogenic Panton–Valentine leucocidin toxin (PVL), which destroys white blood cells and is an important virulence factor [50]. They may be susceptible to a wider range of anti-staphylococcal antibiotics (some are resistant only to beta-lactams). Populations at increased risk for CA-MRSA are listed below [49]:

- Children < 2 years old.
- Athletes (mainly contact-sport participants).
- Injection drug users.
- Homosexual males.
- Military personnel.
- Inmates of correctional facilities, residential homes, or shelters.
- Vets, pet owners, and pig farmers.
- Patients with post-flu-like illness and/or severe pneumonia.
- Patients with concurrent SSTI.
- History of colonization or recent infection with CA-MRSA.
- History of antibiotic consumption in the previous year, particularly quinolones, or macrolides.

MRSA poses a significant and enduring problem to the treatment of infection by such strains. Resistance is usually conferred by the acquisition of a non-native gene encoding a penicillin-binding protein (PBP2a), with significantly lower affinity for beta-lactams. This resistance allows cell-wall biosynthesis, the target of beta-lactams, to continue even in the presence of typically inhibitory concentrations of antibiotic. PBP2a is encoded by the *mecA* gene, which is carried on a distinct mobile genetic element (SCC*mec*). These genetic elements contain two required components: the *mec* gene complex and the *ccr* gene complex (which contains site-specific recombinase genes). The SCC *mec* elements have been classified into eight types (I–VIII) based on the structure and combination of *mec* and *ccr* gene complexes present. These elements also differ in what other antimicrobial resistance genes are carried on them. Types I, IV, V, VI, and VII generally do not carry other resistance genes. Types II, III, and VIII may contain one or more other resistance genes, such as *ermA* (erythromycin), *aadD* (tobramycin), and *tetK* (tetracycline). These types are also used to help distinguish CA-MRSA and HA-MRSA strains. Most HA-MRSA strains carry SCC *mec* types I, II, III, VI, and VIII; while most CA-MRSA strains carry types IV, with some carrying types V and VII [51]. If MRSA is suspected (both HA and CA-MRSA), glycopeptides and other antimicrobial options are available agents. Also, new options such as dalbavancin and tedizolid also can be administered.

## What oral antibiotics can be used for the management of MRSA skin and soft-tissue infections (SSTIs)? What intravenous antibiotics can be used for the management of MRSA skin and soft-tissue infections?

**For oral antibiotic coverage of MRSA in patients with SSTI, we suggest the following agents: linezolid (recommendation 1A), trimethoprim-sulfamethoxazole (TMP-SMX) (recommendation 1B), a tetracycline (doxycycline or minocycline) (recommendation 1B), or tedizolid (Recommendation 1A).**

**For intravenous (IV) antibiotic coverage of MRSA in patients with SSTI, we suggest the following agents: daptomycin (10 mg/kg/dose IV once daily) (recommendation 1A), IV linezolid (recommendation 1A), IV ceftaroline (recommendation 1A), IV dalbavancin (recommendation 1A), IV vancomycin (recommendation 1A), IV tigecycline (recommendation 1A), or IV tedizolid (recommendation 1A).**

**Seven to 14 days of therapy is recommended but should be individualized on the basis of the patient’s clinical response** **(recommendation 1A).** **IV to oral switch should occur when criteria of clinical stability have been reached (recommendation 1C).**

For CA-MRSA, recommended oral agents are clindamycin, although clindamycin resistance is now very common [52], tetracyclines, TMP-SMX, linezolid, tedizolid, and occasionally, fluoroquinolones. Several observational studies and one small randomized trial [53, 54] suggest that TMP-SMX, doxycycline, and minocycline are effective for such infections. If coverage for both streptococci and MRSA is desired for oral therapy, options include clindamycin alone, or the combination of either TMP-SMX or doxycycline with a beta-lactam (e.g., penicillin, cephalexin, or amoxicillin).

Glycopeptides have been for many years the microbiological agents of choice used in complicated Gram-positive infections. Fortunately, staphylococcal resistance to glycopeptides remains rare, although rising minimal inhibitory concentrations (MICs) of glycopeptides may affect the efficacy of these antibiotics [55, 56].

Increased resistance to glycopeptides has encouraged the development of new agents active against Gram-positive bacteria, particularly for severe soft-tissue infections where aggressive antimicrobial management is always recommended, such as linezolid and daptomycin. Linezolid has been considered an agent of choice in complicated skin and soft-tissue infections (cSSTIs). It has the advantages of early intravenous-to-oral switch with the oral preparation having very high bioavailability and excellent tissue penetration [57].

In 2010, an open-label study compared oral or intravenous linezolid with intravenous vancomycin for treatment of cSSTIs caused by MRSA [58]. Patients receiving linezolid had a significantly shorter length of stay and duration of intravenous therapy than those receiving vancomycin. Both agents were well tolerated. Adverse events were similar to each drug’s established safety profile [58].

Recently, a Cochrane meta-analysis included all randomized controlled trials comparing linezolid with vancomycin in the treatment of SSTIs [59]. Linezolid was associated with a significantly better clinical (risk ratio [RR] = 1.09, 95% CI, 1.03–1.16) and microbiological cure rate in adults (RR = 1.08; 95% CI, 1.01–1.16). For infections caused by MRSA, linezolid was significantly more effective than vancomycin in clinical (RR = 1.09; 95% CI, 1.03–1.17) and microbiological cure rates (RR = 1.17; 95% CI, 1.04–1.32).

The daily cost of outpatient therapy was less with oral linezolid than with intravenous vancomycin. Although inpatient treatment with linezolid cost more than inpatient treatment with vancomycin per day, the median length of hospital stay was 3 days shorter with linezolid.

Daptomycin has proven efficacy in patients with Gram-positive complicated ©SSTIs, including those caused by *S. aureus* resistant to methicillin [60]. Daptomycin has been shown to achieve very good concentrations in the skin and soft tissues. In 2010, a meta-analysis compared effectiveness and toxicity of daptomycin with that of other antimicrobials for the treatment of SSTIs. Four studies were included in the analysis (three were randomized RCTs). Vancomycin and semisynthetic penicillins were used in the comparator arm. Three studies reported on patients with cSSTIs. Daptomycin tissue penetration supports its use in the treatment of cSSTIs, and it has been shown to be non-inferior to vancomycin and semisynthetic penicillins [61].

Ceftaroline is an oxyimino advanced-generation broad-spectrum cephalosporin which has in vitro activity against *S. aureus* and MRSA, both of which are associated with cSSTIs. Ceftaroline fosamil has been found to be effective in the treatment of cSSTI when compared with vancomycin plus aztreonam for the treatment of cSSTI [62–64]. Ceftaroline fosamil was also well tolerated and had a safety profile concordant with other antibiotics in the cephalosporin class.

More recently, new drugs have been approved for ABSSSI and have an important activity against MRSA, especially dalbavancin and tedizolid. Tedizolid, a novel oxazolidinone with Gram-positive activity including MRSA, is promising because it can be administered daily in oral or intravenous forms [65, 66], and dalbavancin, a second-generation lipoglycopeptide that covers MRSA, can be administered as infrequently as once weekly [67, 68].

Antibiotics recommended for MRSA infections are listed below.

*Oral options*:

- Minocycline100 mg q12h
- Trimethoprim and sulfamethoxazole 160/800 mg q12h
- Doxycycline 100 mg q12h
- Clindamycin 300–600 mg q8h (high resistance rate)
- Linezolid 600 mg q12h
- Tedizolid 200 mg q24 h

*Intravenous options:*

- Vancomycin 15 mg/kg IV q12h
- Teicoplanin LD 12 mg/kg IV q12h for 3 doses, then 6 mg/kg q12h
- Tigecycline 100 mg IV as a single dose, then 50 mg IV q12h
- Linezolid 600 mg q12h
- Daptomycin 4–6 mg/kg q24h
- Ceftaroline 600 mg q12h
- Dalbavancin 1000 mg once followed by 500 mg after 1 week or 1500 mg one dose
- Tedizolid 200 mg q24h

The decision to use intravenous or oral agents has been debated. Currently, oral therapy is recommended for mild infections and intravenous therapy for severe infections. Moderate infections may be treated via the oral route, or with one to two intravenous doses and then transitioning to oral therapy. For patients with severe infections who are able to tolerate oral therapy and in whom clinical improvement has been documented, the goal should be to transition to the oral route as soon as possible. There is evidence to suggest that this approach positively impacts length of stay as well [69].

## What are necrotizing infections?

Necrotizing soft-tissue infections (NSTIs) are life-threatening, invasive, soft-tissue infections with a necrotizing component involving any or all layers of the soft-tissue compartment, from the superficial dermis and subcutaneous tissue to the deeper fascia and muscle. The latter is most commonly called “necrotizing fasciitis” [70].

**The necrotizing or non-necrotizing character of the infection should be always specified when classifying patients with soft-tissue infections (recommendation 1C).**

Delay in diagnosis and delay in treatment of these infections increase the risk of mortality. Because of its aggressive character, NSTIs should always be differentiated from non-necrotizing infection. Several definitions were published over the last few years, and all these definitions can be confusing. NSTIs are defined by the presence of a spreading infection in any of the layers of the soft tissues (skin, subcutaneous tissue, superficial fascia, deep fascia, or muscles) which is associated with the presence of necrosis of the layer(s) involved and hence requires surgical debridement. All NSTIs fulfill this definition and have common features in their clinical presentation and diagnosis, and most importantly, all of these infections by definition require surgical debridement. Therefore, labeling them with different terms does not serve a useful purpose and may in fact complicate management by delaying diagnosis and/or delaying surgical debridement [7, 8, 10].

## How can necrotizing infections be classified?

**Patients with NSTIs should be classified into the following:**

- **High risk of poor outcome.**
- **Mild/moderate risk of poor outcome.**

**Scores used for severity assessment of patients with necrotizing infections may be useful in the emergency room or outside the intensive care unit (ICU) and may identify patients early, who require surgical treatment and perioperative intensive care management (recommendation 1C).**

Several classifications of NSTIs have been proposed; however, none are universally accepted. Necrotizing infections have been described according to their anatomical locations (i.e., Fournier gangrene) and the depth of infections: dermal and subcutaneous components (necrotizing cellulitis), fascial component (necrotizing fasciitis), and muscular components (necrotizing myositis). NSTIs may also be classified into three types defined by the bacterial pathogens initiating the infection and their typical clinical characteristics: type 1—polymicrobial, type 2—mono-microbial pathogenic β-hemolytic streptococci or CA-MRSA, and type 3—mono-microbial secondary to a variety of pathogenic bacilli. However, resolution of these nomenclature issues requires a consensus among international infectious disease physicians, surgeons, and intensivists, and probably, these various methods of classification are not clinically useful. Although many specific variations of NSTIs have been described, the initial approach to diagnosis, antimicrobial treatment, and surgical intervention is similar for all forms and identifying those infections needing immediate aggressive management is more important than determining the specific variant.

Delay in diagnosis and/or treatment of NSTIs correlates with a poor outcome, leading to multiple organ failure. Early prognostic evaluation of NSTIs is crucial to assess the severity and decide the aggressiveness of treatment. Necrotizing infections remain an important source of patient morbidity and are frequently associated with poor clinical prognosis. Any process of improving quality of care for necrotizing infections globally should focus on simple diagnostic criteria based on physical examination findings and recognition of patients needing timely critical care.

A classification of patients based on a severity assessment which could identify cases requiring surgical and critical care may be an important tool both in ICU and outside of the ICU. The Laboratory Risk Indicator for Necrotizing Fasciitis (LRINEC) score first published in 2004 [71] is based on routinely performed parameters and offers a method to identify NSTIs at early stage. With a score of 8 or higher, there is a 75% risk of a NSTI.

A systematic review of English-language literature from 2004 to 2014 to identify articles reporting use of LRINEC score and the incidence of necrotizing fasciitis was recently published [72]. After application of inclusion criteria, 16 studies with 846 patients were included. The authors concluded that the LRINEC score is a useful clinical determinant in the diagnosis and surgical treatment of patients with necrotizing fasciitis, with a statistically positive correlation between LRINEC score and a true diagnosis of necrotizing fasciitis. A second meta-analysis including English-language studies reporting the diagnostic accuracy of LRINEC score was recently published [73]. Twenty-three studies (*n* = 5982) were included. LRINEC ≥ 6 had sensitivity of 68.2% and specificity of 84.8%, while LRINEC ≥ 8 had sensitivity of 40.8% and specificity of 94.9%. The authors concluded that due to poor sensitivity, LRINEC should not be used to rule-out NSTIs.

In the setting of such aggressive infections, a feasible, low-cost method of rapidly identifying patients requiring critical care is crucial [74]. Early warning system scores utilize physiological, easy-to-measure parameters, assessing physiological parameters such as systolic blood pressure, pulse rate, respiratory rate, temperature, oxygen saturation, and level of consciousness. They are simple, non-invasive, and easy-to-repeat measurement at the bedside.

The Sepsis-3 definitions [75] suggest that patients with at least two of these three clinical variables may be prone for the poor outcome typical of sepsis: (1) low blood pressure (systolic blood pressure ≤ 100 mmHg), (2) high respiratory rate (≥ 22 breaths per minute), or (3) altered mentation (Glasgow coma scale < 15) (quick SOFA [qSOFA]). It is supposed to be useful in out-of-hospital, emergency surgery, or general hospital ward settings, and in patients with positive qSOFA, organ dysfunction should be investigated. The qSOFA score should not be regarded as a diagnostic criterion for defining sepsis. Rather, it should be regarded as a warning for patients with suspected infection who are likely to have poor outcomes. In the setting of patients with necrotizing infections, it may be useful as a warning for patient’s severity assessment.

## Is a multidisciplinary approach to necrotizing infections mandatory?

**A multidisciplinary team is mandatory for the management of NSTIs. Depending on the time line, various specialties are involved. Specific attention should be given to the long-term management of these patients (recommendation 1C).**

NSTIs rank among one of the more difficult disease processes encountered by physicians. The most critical factors for reducing mortality in NSTIs are early recognition and urgent operative debridement. Initial treatment of patients with necrotizing infections should always require coordination between the surgeons, intensivists, and infectious disease specialist. Treatment consists of radical debridement associated with broad-spectrum antimicrobial therapy and hemodynamic support.

Moreover, the magnitude of necrotic tissues that need to be radically debrided, although required to save the patient’s life, often create unique and difficult challenges in terms of wound care, preservation of function, reconstruction, and cosmesis. These problems require time and a multidisciplinary approach. After an extended hospitalization, multiple dressing changes, and surgical procedures, the survivor of NSTI faces months of continued physical therapy to regain functional independence, whenever possible. Rehabilitation is an essential and integral component of recovery [76].

## What is the pathophysiology of necrotizing infections

**Due to the rapid progression of the inflammatory process, early treatment of necrotizing infection is always recommended (recommendation 1C).**

There are two main ways by which bacteria can invade soft tissues. The most common way is through a break in the skin barrier. In case of contamination by spores of *C. perfringens*, the anaerobic environment (caused by impairment of the blood supply resulting in tissue hypoxia) is necessary for maturation and proliferation of *Clostridium* strains [77]. The second way is hematogenous spread of bacteria to the tissue; however, it is a rare condition. Local and systemic manifestations are related to specific pathophysiologic mechanisms depending upon the toxins and enzymes of involved bacteria.

Bacteria proliferate and release toxins, which cause local tissue damage and impair inflammatory responses. Some toxins produce thrombosis of larger venules and arterioles, with subsequent ischemic necrosis of all tissue layers, from the dermis to the deep muscles [77]. Systemic manifestations are also related to toxin-mediated pathophysiologic mechanisms and include fever, hypotension, tachycardia, altered mental status, and signs of organ dysfunction. In principle, these mechanisms may involve both host and pathogen factors. Host-related factors are determined by human genes that control release of cytokines and encode pro-inflammatory cytokines eliciting subsequent counter-regulatory mechanisms. Microbial virulence factors include Gram-positive and Gram-negative bacterial products. These toxins are absorbed in the bloodstream. Bacterial superantigens (pyrogenic exotoxins) directly stimulate and non-specifically activate high numbers of T cells and macrophages to produce pro-inflammatory mediators such as TNF-α, IL-1, and IL-6. The massive release of these cytokines produces an uncontrolled systemic inflammatory response that can lead to multisystem organ dysfunction and shock [77].

## How can necrotizing infections be diagnosed?

**Clinical signs of NSTI include pain out of proportion, edema extending beyond the erythema, and fever.** **A rapidly progressive soft-tissue infection should always** **be suspected as a necrotizing infection** **(recommendation 1C)**.

The initial differential diagnosis between a cellulitis and a necrotizing infection that requires prompt operative intervention may be difficult. Most cases of NSTI are initially diagnosed and begin as cellulitis. However, since time to operative debridement is an important determinant of outcome in necrotizing infections, timely diagnosis is essential.

Patients with NSTI usually present with severe pain which is out of proportion to the physical findings [78–82].

Typical local signs are as follows:

- Edema
- Erythema
- Severe and crescendo pain out of proportion
- Skin bullae or necrosis (at later stage)
- Swelling or tenderness
- Crepitus

Systemic signs are as follows:

- Fever
- Tachycardia
- Hypotension
- Shock

Laboratory tests are not highly sensitive or specific for NSTIs. A rapidly progressive soft-tissue infection should be treated as a necrotizing infection, from the beginning. The clinical picture may worsen very quickly, sometimes during a few hours.

In order to predict the presence of NSTI, the Laboratory Risk Indicator for Necrotizing infection (LRINEC) score was proposed [71]. LRINEC score assigns points for abnormalities in six independent variables: serum C-reactive protein level (> 150 mg/L), white blood cell (WBC) count (> 15,000/μL), hemoglobin level (< 13.5 g/dL), serum sodium level (< 135 mmol/L), serum creatinine level (> 1.6 mg/dL [142 mmol/l]), and serum glucose level (> 180 mg/dL [10 mmol/l]). With a score of 8 or higher, there is a 75% risk of a NSTI.

Subsequent evaluation of the LRINEC score has demonstrated conflicting results. Several studies have assessed the utility of LRINEC for the early diagnosis of necrotizing infections [72, 83–88].

Recent evidence has demonstrated that it lacks the sensitivity to be a useful adjunct for the diagnosis of necrotizing infections [73].

**The diagnosis of necrotizing infection is primarily a clinical diagnosis. However, radiologic imaging may be able to provide useful information when the diagnosis is uncertain. A plain X-ray should not be used to rule-out necrotizing infection (recommendation 1B).**

**In unstable patients, ultrasound may be useful to differentiate simple cellulitis from necrotizing fasciitis (recommendation 2C).**

**Imaging studies should not delay surgical consultation and intervention (recommendation 1A).**

Frequently, plain radiographs are normal or with increased soft-tissue thickness and opacity, unless the infection and necrosis are advanced. The characteristic finding is gas in the soft tissues, but subcutaneous gas is present only in few cases of necrotizing infection and is not present in pure aerobic infections such as those caused by *S. pyogenes*.

Additionally, subcutaneous gas may not be present in earlier stages of the disease process and only become manifest as the patient’s condition deteriorates [89, 90].

Computed tomography (CT) has a higher sensitivity than plain radiography in identifying early NSTIs. Findings consistent with necrotizing infections are fat stranding, fluid and gas collections that dissect along fascial planes, and gas in the involved soft tissues. Additionally, fascial thickening and non-enhancing fascia on contrast CT suggests fascial necrosis [91].

In 2010, a case series study [92] analyzing the use of CT scanning for the diagnosis of NSTIs was published. Of 67 patients with study inclusion criteria, 58 underwent surgical exploration, and NSTIs was confirmed in 25 (43%). The remaining 42 patients had either non-necrotizing infections during surgical exploration (*n* = 33) or were treated non-operatively with successful resolution of the symptoms (*n* = 9). The sensitivity of CT to identify NSTI was 100%, specificity was 81%, positive predictive value was 76%, and negative predictive value was 100%.

Magnetic resonance imaging (MRI) has been the imaging modality of choice for necrotizing fasciitis. Patients with necrotizing fasciitis usually have a significantly greater frequency of the following MRI findings: thick (≥ 3 mm) abnormal signal intensity on fat-suppressed T2-weighted images, low signal intensity in the deep fascia on fat-suppressed T2-weighted images, a focal or diffuse non-enhancing portion in the area of abnormal signal intensity in the deep fascia, extensive involvement of the deep fascia, and involvement of three or more compartments in one extremity [93]. However, MRI may be difficult to perform under emergency conditions and is not recommended as the first-choice imaging technique.

Ultrasound has the advantage of being rapidly performed at bedside and may be helpful in differentiating simple cellulitis from necrotizing fasciitis. In a prospective observational study of 62 patients with clinically suspected necrotizing fasciitis, ultrasound had a sensitivity of 88.2%, specificity of 93.3%, positive predictive value of 95.4%, negative predictive value of 95.4%, and diagnostic accuracy of 91.9%. The authors considered the findings of diffuse subcutaneous thickening accompanied by fluid accumulation of > 4 mm in depth along the deep fascial layer predictive of necrotizing fasciitis [94].

Rapid performance of frozen-section soft-tissue biopsy, early in the evolution of a suspect lesion, may provide a definitive and life-saving diagnosis. Triple diagnostics which include an incisional biopsy over the most suspected area, a fresh frozen section and Gram staining might be an important adjunct in early stages of suspected necrotizing infections (recommendation 1C).

Early frozen-section diagnosis should be limited to those cases in which the clinical or radiographic findings are not diagnostic (recommendation 1C).

Fascial biopsy with frozen section has been suggested as a means to achieve earlier diagnosis of NSTIs [95, 96]. However, frozen-section biopsy is not very practical and requires availability and experience of the pathologists, and the time taken to carry out and analyze the sample could be used for debridement [97]. The Finger test is another adjunct method described for diagnosing NSTIs. It is performed under local anesthesia. A 2-cm incision is made down to the deep fascia. Minimal tissue resistance to finger dissection (positive Finger test), the absence of bleeding, presence of necrotic tissue, and/or murky and grayish (“dishwater”) fluid following incision, all suggest the diagnosis of NSTI [98].

## What is the best timing of source control?

**Provide (surgical) source control in patients with NSSTI as soon as possible, but at least within the first 12 h after admission, in patients with a high suspicion for necrotizing infection. Early source control, antimicrobial therapy, and (organ) supportive measures are the cornerstone of treatment in patients with sepsis or septic shock caused by NSSTI (recommendation 1B).**

Source control for SSTIs includes drainage of infected fluids, debridement of infected soft tissues, and removal of infected devices or foreign bodies. It should also include definitive measures to correct any anatomic derangement resulting in ongoing microbial contamination and restoring optimal function. Early surgical debridement with complete removal of necrotic tissue is essential to decrease mortality and other complications in patients with NSTIs. It is the most important determinant of outcome in necrotizing infections. This was well described in a study by Bilton et al. in which patients with NSTIs, who had adequate surgical debridement (early and complete), were compared to those with either delayed or incomplete debridements. The mortality in the latter group was 38% compared to 4.2% in the group receiving early adequate surgical treatment [99].

Delay in source control in patients with NSTIs has been repeatedly associated with a greater mortality. A retrospective study including 47 patients with the diagnosis of NSTI admitted to a large academic hospital from December 2004 to December 2010 was published in 2011 [100]. Overall mortality was 17.0%. The average number of surgical debridements in patients with surgical treatment delayed > 12 h from the time of emergency department admission was significantly higher than those who had an operation within 12 h after admission (7.4 ± 2.5 versus 2.3 ± 1.2; *p* < 0.001). Delayed surgical debridement was associated with significantly higher mortality, higher incidence of septic shock and renal failure, and more surgical debridements than patients with early surgical debridements. After adjusting for possible confounding factors, the average number of surgical debridements and the presence of septic shock and acute renal failure were still significantly higher in patients in whom surgery was delayed > 12 h.

A retrospective study including 106 patients with necrotizing infections conducted in a medical ICU was published in 2009 [101]. Overall hospital mortality was 40.6%. In multivariate analysis, underlying cardiovascular disease, SAPS II, abdomino-perineal compared to limb localization, time from the first signs to diagnosis < 72 h, and time from diagnosis to surgical treatment > 14 h in patients with septic shock were independently associated with hospital mortality.

A retrospective study of 121 patients (mean age, 65.2 ± 11.6 years) with *Vibrio vulnificus*-related necrotizing infection who underwent surgical intervention between July 1998 and June 2011 was published in 2011 [102]. The patients were divided into three groups according to the time between admission and surgical treatment as follows: surgical treatment less than 12 h after admission, 12 to 24 h after admission, and more than 24 h after admission. Patients who underwent surgery less than 12 h after admission had a significantly lower mortality compared with those who had surgery either 12 to 24 h after admission (adjusted hazard ratio [HR], 0.064; 95% CI, 1.6 × 10^−7^ to 0.25; *p* = 0.037) or more than 24 h after admission (adjusted HR, 0.0043; 95% CI, 2.1 × 10^−5^ to 0.0085; *p* = 0.002). There was no difference in mortality risk between patients who underwent surgery 12 to 24 h after admission and those who had surgery more than 24 h after admission (*p* = 0.8).

We suggest to remove only devitalized/infarcted skin and spare normally perfused skin. In case where skin viability is questionable, skin preservation and reassessment at the second operation is indicated (recommendation 1C).

Once the decision to take the patient for an operation has been made, the initial incision is done in the compromised area and the wound is explored for macroscopic findings of NSTIs. Incision should take place along the involved muscular lodges. Removal of all non-viable tissue should be accomplished including muscle, fascial layers, subcutaneous tissue, and skin if they are compromised, and one should extend the incision until healthy viable tissue is seen. Removal of previously viable skin or muscle should usually not be done at the initial operation, attempting skin sparing via multiple incisions (to preserve perforators), while accounting for underlying bone, nerve, and vascular structures. Skin perfusion and viability can easily be assessed at re-exploration, and removal at that time is easy, if indicated. The wound should always be left open. Amputation of a limb does not add to the acute debridement and should be reserved for late and extreme presentations.

## What is the best timing of re-exploration?

**Consider to plan the first re-exploration within 12–24 h and to repeat re-exploration(s) until the patient is free of necrosis (recommendation 1C).**

There is a lack of literature examining outcomes in necrotizing infections when surgical re-debridements are performed in early versus delayed intervals. Scheduled re-explorations should be done at least every 12–24 h after the initial operation or sooner if clinical local or systemic signs of worsening infection become evident, as well as with worsening laboratory parameters (particularly WBC count). Re-explorations should be repeated until the time when very little or no debridement is required.

A prospective observational study by Okoye et al. [103] showed that delayed re-debridement after initial source control in necrotizing infections results in worse survival and an increased incidence of acute kidney injury. The authors concluded that further studies to identify the optimal time interval for re-debridement are warranted.

## What is the role of hyperbaric oxygen therapy (HBO) for source control in necrotizing infections?

**Consider adjuvant hyperbaric oxygen therapy in patients with NSTI after prompt debridement (recommendation 2B).**

Despite significant advancements in critical care management as well as improved knowledge regarding NSTIs, mortality remains relatively high. Adjunctive and less conventional treatment options have been explored in an effort to improve outcomes in this group of patients. Hyperbaric oxygen (HBO) is one of these modalities. It is a medical treatment that uses delivery of 100% oxygen at a pressure of 2–3 absolute atmospheres. Its use is motivated by the fact that oxygen delivery at these parameters achieves a much higher concentration of dissolved oxygen in blood which results in higher tissue oxygen tensions. At this higher tissue tension, beneficial effects may be seen including improved leukocyte function, inhibition of anaerobic growth, inhibition of toxin production, and enhancement of antibiotic activity.

The role of HBO as an adjunctive treatment has been debated, and no prospective randomized clinical trials have been published. In order to determine the effect of hyperbaric oxygen HBO therapy on mortality, complication rate, discharge status/location, hospital length of stay, and inflation-adjusted hospitalization cost in patients with NSTIs, a retrospective study of 45,913 patients in the Nationwide Inpatient Sample from 1988 to 2009 was published in 2012 [104]. This retrospective analysis of HBO therapy in NSTI showed that despite the higher hospitalization cost and longer length of stay, the statistically significant reduction in mortality supports the use of HBO therapy in NSTI (RR = 0.47; 95% CI, 0.30–0.74). In 2013, a review about HBO therapy for treating acute surgical and traumatic wounds was published [105]. The authors concluded that there is a lack of high-quality, valid research evidence regarding the effects of HBO therapy on wound healing.

HBO could be useful, if available, but it should not interfere with the standard treatment. Furthermore, the patient should not be transferred to carry out HBO therapy, thereby delaying standard care.

## What is the role of intravenous immunoglobulin (IVIG) therapy for source control in necrotizing infections?

**Consider intravenous immunoglobulin (IVIG) therapy in patients with necrotizing infections caused by GAS (recommendation 2B).**

Intravenous immunoglobulin therapy has been postulated to improve outcomes in a selected population of patients with NSTIs. Most of the reported studies evaluated its use for invasive GAS infections including GAS-related NSTIs with streptococcal toxic shock syndrome (STSS).

The efficacy and safety of high-dose IVIG as adjunctive therapy in STSS were evaluated in a multicenter, randomized, double-blind, placebo-controlled trial [106]. The trial was prematurely terminated because of slow patient recruitment, and results were obtained from 21 enrolled patients (10 IVIG recipients and 11 placebo recipients). The primary end point was mortality at 28 days, and a 3.6-fold non-significant higher mortality rate was found in the placebo group. A significant decrease in the sepsis-related organ failure assessment score at days 2 (*p* = 0.02) and 3 (*p* = 0.04) was noted in the IVIG group. Furthermore, a significant increase in plasma neutralizing activity against superantigens expressed by autologous isolates was noted in the IVIG group after treatment (*p* = 0.03). Although statistical significance was not reached in the primary end point, the trial provides some supportive evidence for IVIG as an efficacious adjunctive therapy in STSS. IVIG therapy role in improving survival in STSS was also demonstrated in other prospective studies [107, 108].

In 2017, a retrospective study of adult patients with necrotizing fasciitis and vasopressor-dependent shock undergoing surgical debridement from 2010 to 2014 in 130 US hospitals was published [109]. Of 4127 cases of debrided necrotizing infection with shock at 121 centers, only 164 patients (4%) at 61 centers received IVIG. IVIG subjects were younger with lower comorbidity indices, but higher illness severity. Clindamycin and vasopressor intensity were higher among IVIG cases, as was coding for TSS and GAS. In-hospital mortality did not differ between matched IVIG and non-IVIG groups (crude mortality, 27.3 versus 23.6%; adjusted HR, 1.00 [95% CI, 0.55–1.83]; *p* = 0.99). Early IVIG (≤ 2 days) did not alter this effect (*p* = 0.99). Among patients coded for TSS, GAS, and/or *S. aureus*, IVIG use was still unusual (6.8%) and lacked benefit (*p* = 0.63). Median LOS was similar between IVIG and non-IVIG groups (26 [13–49] versus 26 [11–43]; *p* = 0.84).

Recently, a Cochrane review on intervention for NSTIs was published [110]. One trial of 100 randomized participants assessed IVIG as an adjuvant drug, given at a dose of 25 g/day, compared with placebo, given for three consecutive days. No clear difference between IVIG and placebo in terms of mortality within 30 days (RR = 1.17; 95% CI, 0.42–3.23), nor serious adverse events experienced in the ICU (RR = 0.73; 95% CI, 0.32–1.65) were observed.

## What are the resuscitation principles in patients with necrotizing infection?

**Supportive treatment in managing necrotizing infections must be early and aggressive to halt progression of the inflammatory process (recommendation 1A).**

Early detection of sepsis and prompt aggressive treatment of the underlying organ dysfunction is an essential component for improving outcomes of critical ill patients [111]. Necrotizing infections may present with a fulminant course and may be associated with great morbidity and high case-fatality rates, especially when they occur in conjunction with TSS.

Early blood cultures, empirical antibiotic treatment, and intensive care for hemodynamic and metabolic support should be performed as soon as possible. Moreover, patients may lose fluids, proteins, and electrolytes through a large surgical wound [112]. In addition, hypotension is caused by vasodilation induced by the systemic inflammatory response syndrome to infection [113]. Fluid resuscitation and analgesia are the mainstays of support for patients with advanced sepsis usually combined with vasoactive amines associated with mechanical ventilation and other organ function supports, if needed. No ideal fluid exists: resuscitation therapy must be prompt and immediate as in any type of shock.

## What are the new agents to treat necrotizing infections

**AB103 (Reltecimod) is a new agent for modulation of inflammation after necrotizing infections. Further study is warranted to establish efficacy (no recommendation).**

AB103 (Reltecimod) is a safe and promising new agent for modulation of inflammation after a necrotizing infections. However, further studies are warranted to establish efficacy.

AB103 (originally p2TA) is a novel synthetic CD28 mimetic octapeptide that selectively inhibits the direct binding of superantigen exotoxins to the CD28 costimulatory receptor on T-helper 1 lymphocytes [114].

Preclinical studies demonstrated that AB103 and related superantigen mimetic peptides are associated with improved survival in animal models of toxic shock and sepsis. The hypothesis is that AB103 could be administered safely in patients presenting with NSTI and would modulate the immune response to reduce the development or progression of organ failure.

To establish the safety of AB103 in patients with NSTI and evaluate the potential effects on clinically meaningful parameters related to the disease [115], a prospective, randomized, placebo-controlled, double-blinded study was performed in six academic medical centers in the USA. Participants included adults with NSTI. Of 345 patients screened, 43 were enrolled for the intent-to-treat analysis, and 40 met criteria for the modified intent-to-treat analysis; 15 patients each were included in the high-dose and low-dose treatment arms, and 10 in the placebo arm. Baseline characteristics were comparable in the treatment groups. The Sequential Organ Failure Assessment score improved from baseline in both treatment groups compared with the placebo group at 14 days (change from baseline score, − 2.8 in the high-dose, − 2 in the low-dose, and + 1.3 in the placebo groups; *p* = 0.04). AB103-treated patients had a similar number of debridements (mean [SD], 2.2[1.1] for the high-dose, 2.3[1.2] for the low-dose, and 2.8 [2.1] for the placebo groups; *p* = 0.56). There were no statistically significant differences in ICU-free and ventilator-free days or in plasma and tissue cytokine levels. No drug-related adverse events were detected. A phase 3 trial, also known as the ACCUTE trial (Reltecimod Clinical Composite Endpoint Study in Necrotizing Soft Tissue Infections), has been designed as a single pivotal study to assess the efficacy and safety of Reltecimod versus placebo in patients with necrotizing infections.

## What antibiotics are recommended for empiric treatment of clinically suspected necrotizing infections?

**Antibiotic treatment of necrotizing infections should be prompt and aggressive (recommendation 1B).**

**The initial empirical antibiotic regimen should comprise broad-spectrum drugs including anti-MRSA and anti-Gram-negative coverage (recommendation 1C).**

**Vancomycin treatment should be avoided in patients with renal impairment and when MRSA isolate shows a MIC for vancomycin ≥ 1.5 mg/mL (recommendation 1B).**

**Daptomycin or linezolid are drugs of choice for empirical anti-MRSA coverage. Alternatively, ceftaroline, telavancin, tedizolid, and dalbavacin can be used (recommendation 2C).**

**The choice of anti-Gram-negative treatment should be based on local prevalence of ESBL-producing** ***Enterobacateriaceae*** **and multidrug-resistant organisms (MDROs) non-fermenters (recommendation 1B).**

**De-escalation of antibiotic therapy should be based on clinical improvement, cultured pathogens, and results of rapid diagnostic tests where available (recommendation 1C).**

Microbiologically, NSTIs have been classified as either type 1 (polymicrobial) or type 2 (mono-microbial) or type 3 (gas gangrene). Occasionally in immunocompromised patients, NSTIs may be also caused by mycotic species.

NSTIs type I is a polymicrobial infection involving aerobic and anaerobic organisms. It is usually seen in the elderly or in those with underlying illnesses [77]. Type I infection is often associated with gas in the tissue and thus is difficult to distinguish from gas gangrene. Non-clostridial anaerobic cellulitis and synergistic necrotizing cellulitis are type I variants. Both occur in patients with diabetes and typically involve the feet, with rapid extension into the leg.

NSTI type II is a mono-microbial infection. Among Gram-positive organisms, GAS remains the most common pathogen, followed by MRSA. Unlike type I infections, type II infections may occur in any age group and in persons without any underlying illness. Other pathogens include *Aeromonas hydrophila* and *V. vulnificus*. Mono-microbial necrotizing fasciitis due to Gram-negative pathogens (bacteroides and *E. coli*) have also been reported, though these infections are typically seen in immunocompromised, diabetic, obese, and postoperative patients [77].

Gas gangrene (clostridial myonecrosis), or type III NSTI, is an acute infection by clostridium or bacillus of healthy living tissue that occurs spontaneously or as a result of traumatic injury. Recurrent gas gangrene, occurring several decades after the primary infection, has also been described.

The use of antimicrobial therapy is an adjuvant treatment and must be combined with early surgical debridement. Once the diagnosis is made and blood cultures have been drawn, broad-spectrum coverage should be urgently commenced. Initial antibiotic therapy for necrotizing infections is empirical in nature because microbiological data (culture and susceptibility results) may require > 24 h before they are available for a more detailed analysis.

Since it is impossible to exclude with certainty a polymicrobial necrotizing infection, an aggressive broad-spectrum empiric antimicrobial therapy should initially be selected to cover Gram-positive, Gram-negative, and anaerobic organisms until culture-specific results and sensitivities are available. An acceptable empiric antibiotic regimen should always include antibiotics, which cover MRSA with the additional benefit of inhibiting invasive GAS virulence proteins. For the treatment of MRSA, we refer to the previous paragraphs.

For the treatment of Gram-negative bacteria, the use of piperacillin-tazobactam in the setting without high local prevalence of ESBL-producing *Enterobacateriaceae* optimizing pharmacokinetic*/*pharmacodynamic parameters is appropriate*.* Carbapenems, administered in adequate dosage, including meropenem, imipenem-cilastatin, or doripenem may be used in the settings with high local prevalence of ESBL-producing *Enterobacateriaceae*. Culture-specific results and sensitivities can direct both broadening of antimicrobial regimen if it is too narrow and a de-escalation if it is too broad particularly in critically ill patients where de-escalation strategy is one of the cornerstones of antimicrobial stewardship programs [116]. The choice of anti-Gram-negative treatment should be based on local prevalence of ESBL-producing *Enterobacateriaceae* and MDRO non-fermenters.

## Should an antitoxin active drug (clindamycin or oxazolidinon) be included in the empirical regiment of clinically suspected necrotizing infection?

**Either clindamycin or linezolid should be included in the empirical antibiotic regimen of NSTI (recommendation 1C).**

Selection of antibiotics that inhibit toxin production may be helpful, particularly in those patients who have evidence of TSS, potentially present in patients who have streptococcal and staphylococcal infections. Protein cytotoxins play an important role in the pathogenesis of various staphylococcal infections, and toxin production should be considered when selecting an antimicrobial agent for Gram-positive pathogens. Linezolid and clindamycin play an important role because they may significantly inhibit exotoxin production from Gram-positive pathogens [117–119].

## What is the optimal duration of antibiotic therapy for necrotizing infections?

**In the absence of definitive clinical trials, antibiotic therapy should be administered until further debridement is no longer necessary, the patient has improved clinically, and fever has resolved for 48–72 h (recommendation 1C).**

**Procalcitonin monitoring may be useful to guide antimicrobial discontinuation (recommendation 2B).**

There is no direct evidence about optimal duration of antibiotic therapy, and the expert panel shares that antimicrobial therapy should be administered until further debridement is no longer necessary, the patient has improved clinically, and fever has been resolved for 48–72 h.

Several controlled clinical studies have evaluated the potential of the infection biomarker procalcitonin (PCT) to improve the diagnostic work-up of patients with bacterial infections and its influence on decisions regarding antibiotic therapy [120].

In order to develop a PCT ratio indicating successful surgical intervention in patients with necrotizing infections, Friederichs et al. [121] designed a study of 38 patients treated with clinical signs of sepsis caused by a NSTI. All patients received radical surgical treatment. Serum levels of PCT and C-reactive protein were monitored postoperatively. The ratio of day 1 to day 2 was calculated. An eradication of the infectious focus was successfully performed in 84% of patients, averaging 1.9 operations (range 1–6) to achieve the elimination of the infectious source. The PCT ratio was significantly higher in the group of patients with successful surgical intervention (1.665 versus 0.9, *p* < 0.001). A ratio higher than the calculated cutoff of 1.14 indicated successful surgical treatment with a sensitivity of 83.3% and a specificity of 71.4%. The positive predictive value was 75.8%, and the negative predictive value was 80.0%.

The PCT ratio of postoperative day 1 to day 2 following major surgical procedures for necrotizing infections represented a valuable clinical tool indicating successful surgical eradication of the infectious focus and correlated with the successful elimination of the infectious source and clinical recovery.

## What is the treatment of Fournier’s gangrene?

**Treatment of Fournier’s gangrene includes prompt appropriate antibiotic therapy, hemodynamic support, and early debridement (recommendation 1C).**

**Early and extensive initial surgical debridement in Fournier’s gangrene patients improves survival (recommendation 1C).**

**We suggest consideration for fecal diversion—either by colostomy, fecal tube system with or without negative pressure therapy—in cases of Fournier’s gangrene with fecal contamination (recommendation 2C).**

Fournier’s gangrene (FG) is a severe type of NSTI involving the genital area and or perineum. It was initially described by Baurinne in 1764 and is named after Jean Alfred Fournier, a French dermatologist who in 1883 described it. Due to the complexity of fascial planes, the infection may extend up to the abdominal wall, down into the thigh areas, into the perirectal and gluteal spaces, and, occasionally, into the retroperitoneum. Advanced FG can extend through the fascial planes ascending as high as the torso and descending to the thighs. The perineal fascia, Colles’ fascia, is continuous with Scarpa’s fascia of the anterior abdominal wall and Buck and Dartos’ fascia of the penis and scrotum. Testicular involvement is rare, and this has been attributed to their non-perineal blood supply. It has a mortality rate that approaches 20–50% in many contemporary series [122–124]. The origin of the infection is identifiable in the majority of cases and is predominantly from anorectal, genito-urinary, or local cutaneous sources [125]. The aggressive nature of the infection requires early recognition and immediate surgical intervention.

Diagnosis is based on clinical signs and physical examination. Including cutaneous manifestations, erythema, subcutaneous crepitations, patches of gangrene, a presence of potential portal of entry, foul smell, purulence and/or wound discharge, and tenderness to palpitation. Imaging, including conventional radiology, US, CT, and MRI may be used to confirm clinical suspicions and to help in identifying the extent of the soft-tissue involvement, particularly in the perirectal and retroperitoneal planes.

Fournier’s Gangrene Severity Index (FGSI) is a standard score for predicting outcome in patients with FG and is obtained from a combination of physiological parameters at admission including temperature, heart rate, respiration rate, sodium, potassium, creatinine, leukocytes, hematocrit, and bicarbonate. A FGSI score above 9 has been demonstrated to be sensitive and specific as a mortality predictor in patients with Fournier’s gangrene [126, 127].

Surgical debridement must be early and aggressive to halt progression of infection. Cultures of infected fluid and tissues should be obtained during the initial surgical debridement and the results used to tailor specific antibiotic management. Radical surgical debridement of the entire affected area should be performed, continuing the debridement into the healthy-looking tissue [128, 129].

In the setting of FG, diverting colostomy has been demonstrated to improve outcomes. It helps in decreasing sepsis by minimizing bacterial load in the perineal wound, thus controlling infection [130]. Diverting colostomy does not eliminate the necessity of multiple debridements, nor reduces the number of these procedures [131]. Diverting colostomy should be avoided as much as possible mainly when there are other methods to avoid wound contamination. Recently, rectal diversion devices have been marketed. They are silicone tubes designed to divert fecal matter in patients with diarrhea, local burns, or skin ulcers. The devices protect the wounds from fecal contamination and reduce, in the same way a colostomy does, both the risk of skin breakdown and repeated inoculation with colonic microbial flora. Fecal diversion tubes can be used in combination with negative pressure wound therapy (NPWT) for effective isolation of the wound from fecal contamination. Estrada et al. showed that this was an effective way for fecal diversion and constitutes an attractive alternative to colostomy [132].

## What is the role of negative pressure wound therapy in soft-tissue infections and necrotizing fasciitis?

**We suggest to consider negative pressure wound therapy (NPWT) for wound care after complete removal of necrosis in necrotizing infections (recommendation 1C).**

The rapidly spreading infection followed by aggressive surgical intervention and repeated debridements creates challenges for wound management. NPWT refers to wound dressing systems that continuously or intermittently apply sub-atmospheric pressure to the surface of a wound. NPWT has become a popular treatment modality for the management of many acute and chronic wounds. Sub-atmospheric pressure has multiple beneficial effects on wound healing in animal models. Animal and human studies have shown that sub-atmospheric pressure improves the local wound environment through both direct and indirect effects; these effects accelerate healing and reduce the time to wound closure [133].

In the setting of necrotizing infections once the necrosis is removed, NPWT can help wound healing physiologically. The negative pressure leads to an increased blood supply, increasing tissue perfusion, reducing edema, absorbing fluids and exudates, inhibiting infection, and finally drying the wound and thus the migration of inflammatory cells into the wound. Additionally, it promotes and accelerates the formation of granulation tissue by the removal of bacterial contamination and exudates.

Although evidence of promising results with NPWT is increasing in other fields [134–143], in NSTIs, the clinical evidence of its superiority over conventional wound dressing techniques for all wound types has not been proven [144, 145].

A systematic review of PubMed and Cochrane Library databases for randomized, controlled trials (RCTs) of NPWT for the treatment of acute or chronic wounds was published in 2011, which did not find clear evidence of a beneficial effect of NPWT compared with conventional treatment [146]. However, much evidence of a positive effect of NPWT on wound in general has been published since and the 2011 systematic review is outdated.

## What is the treatment of infected meshes?

**Respect prevention strategies to avoid surgical site infection and prosthetic contamination (recommendation 1A).**

**We suggest avoidance of mesh contamination following incisional SSI by an early and adequate local source control as well as antibiotic treatment (recommendation 1C).**

**In chronic sinuses and infected meshes, we suggest a complete surgical removal of the mesh which remains the only way to eradicate infection (recommendation 1C).**

**No clear recommendations on the benefit of biologic versus synthetic mesh in potentially contaminated fields can be proposed (recommendations 1C).**

Hernia repair is one of the most common surgical procedures performed globally. Mesh infection, although infrequent, is a devastating complication of mesh hernioplasties, and for this reason, a prevention strategy is essential. Currently, several types of prosthetic mesh are widely used for repairing abdominal wall defects; however, there is no single universal ideal mesh. Synthetic meshes are easy to handle and well tolerated; however, they can be potentially associated with infection when bacteria adhere to the synthetic material leading to chronic infection. Mesh infection is a challenging complication of abdominal wall defect repairs [147–149].

Polypropylene remains the most commonly used material for hernia repairs. Synthetic meshes consisting of large pore meshes are more resistant to infection than the firm, smaller pore meshes. Although biological meshes cost more than synthetic meshes and the long-term durability may be less favorable [150, 151], they can confer protective factors such as resistance to infection and high biocompatibility when implanted [148].

In order to evaluate the risk factors for mesh-related infections after surgical hernia repair, a systematic search performed in PubMed and Scopus databases was published in 2011 [152]. The crude mesh infection rate was 5%. Statistically significant risk factors were smoking (RR = 1.36 [95% CI 1.07, 1.73]; 1171 hernioplasties), American Society of Anesthesiologists (ASA) score ≥ 3 (RR = 1.40 [1.15, 1.70]; 1682 hernioplasties), and emergency operation (RR = 2.46 [1.56, 3.91]; 1561 hernioplasties). Also, mesh infections were significantly correlated with patient age (weighted mean difference [WMD] = 2.63 [0.22, 5.04]; 2364 hernioplasties), ASA score (WMD = 0.23 [0.08, 0.38]; 1682 hernioplasties), and the duration of the hernioplasty (WMD = 44.92 [25.66, 64.18]; 833 hernioplasties). A trend toward higher mesh infection rates was observed in obese patients (RR = 1.41 [0.94, 2.11]; 2243 hernioplasties) and in patients operated on by a resident (in contrast to a consultant; RR = 1.18 [0.99, 1.40]; 982 hernioplasties). Mesh infections usually resulted in mesh removal, and *Staphylococcus* spp., *Enterococcus* spp., and Gram-negative bacteria were the germs commonly isolated in the specimen.

In 2017, a retrospective review of all patients who underwent abdominal wall hernia repair from January 2004 to May 2014 at a tertiary center was published [153]. From 3470 cases of abdominal wall hernia repair, 66 cases (1.9%) of mesh infection were reported, and 48 of these patients (72.7%) required mesh explantation. Steroid or immunosuppressive drugs use (odds ratio [OR] 2.22; CI 1.16 to 3.95), urgent repair (OR 5.06; CI 2.21 to 8.60), and postoperative surgical site infection (OR 2.9; CI 1.55 to 4.10) were predictive of mesh infection. Independent predictors of mesh explantation were type of mesh (OR 3.13; CI 1.71 to 5.21), onlay position (OR 3.51; CI 1.23 to 6.12), and associated enterotomy in the same procedure (OR 5.17; CI 2.05 to 7.12).

The pathogenesis of mesh infection is a complex process involving many factors including, but not limited to, bacterial virulence, surface physicochemical properties of the prosthetic material, and alterations in host defense mechanisms. The result of this interaction is the formation of the bacterial biofilm. Embedded in self-secreted extracellular polymeric substances, biofilm can provide bacteria an effective barrier against host immune cells and antibiotics [154–156]. Early antibiotics and mechanical scrubbing or irrigation to remove the biofilm before it is consolidated are both important.

Mesh infections should be distinguished from superficial incisional SSIs. They occur in the early postoperative period and are not influenced by mesh implantation but can cause the infection of the mesh. The diagnosis of wound infection is clinical, with typical symptoms of localized inflammation and pain at the incision site. Patients with deep mesh infections may present with signs of local inflammation. However, more frequently, deep mesh infections tend to be indolent and present chronic signs and symptoms. They may be initially underestimated.

The management of mesh-site infections is challenging and always requires an individualized approach combining medical and surgical approaches. Clinical trials have demonstrated that in certain instances, non-operative strategies with conservative (non-surgical) management have been successful for salvaging a mesh [157]. If conservative treatment fails, complete surgical removal of the mesh is suggested to reduce the risk of infection recurrence or severe complications, such as visceral adhesions and fistulae. A conservative surgical approach including abscess drainage, sinus excision, or partial mesh excision can fail and may result in recurrent mesh infections.

After removing the infected mesh, the intra-operative options are (a) no implant of a new mesh, (b) re-implantation of a new synthetic light-weight, macroporous mesh, and (c) replacement of the infected synthetic by a biological mesh [158, 159]. A critical issue in the repair of contaminated abdominal wall defects is the dilemma of choosing between synthetic material, with its presumed risk of surgical site complications, and biologic material, a costly alternative with questionable durability. In 2016, Atema et al. published a systematic review and meta-analysis of the repair of potentially contaminated and contaminated abdominal wall defects [160]. Thirty-two studies published between January 1990 and June 2015 on repair of (potentially) contaminated hernias with ≥ 25 patients were reviewed. Fifteen studies solely described hernia repair with biologic mesh, 6 non-absorbable synthetic meshes, and 11 described various techniques. Studies reporting direct and prospective comparison of synthetic versus biological mesh in a cohort were not found. Surgical site complications and hernia recurrence rates were evaluated per degree of contamination and mesh type by calculating pooled proportions. In potentially contaminated hernias (CDC wound class 2), no benefit of biologic over synthetic mesh was found with comparable surgical site complication rates and a hernia recurrence rate of 9% for biologic and 9% for synthetic repair. In contaminated hernias (CDC wound class 3 and 4), most reports were on biologic mesh repair, showing high rates of surgical site complications and a hernia recurrence rate of 30%. Recurrence rates in contaminated hernias depended on whether primary fascial closure was achieved, or the repair with biologic mesh was bridging. Biologic mesh sublay repair with primary fascial closure showed lower recurrence rates than bridging repairs. Non-cross-linked biologic mesh can be used in contaminated hernia without mesh infection and subsequent need for mesh explantation. As only one study on synthetic repair of contaminated hernias was available in literature, no recommendation can be given on the use of synthetic mesh in this setting [161].

## Conclusions

SSTIs encompass a variety of pathological conditions ranging from simple superficial infections to severe necrotizing infections. The multifaceted nature of these infections has led to a collaboration among general and emergency surgeons, intensivists, and infectious diseases specialists, who have shared these clinical practice recommendations.

## Abbreviations

NSTIs: Necrotizing soft-tissue infections
SSIs: Surgical site infections
SSTIs: Skin and soft-tissue infections
